# What Are Reactive Oxygen Species, Free Radicals, and Oxidative Stress in Skin Diseases?

**DOI:** 10.3390/ijms221910799

**Published:** 2021-10-06

**Authors:** Kozo Nakai, Daisuke Tsuruta

**Affiliations:** Department of Dermatology, Graduate School of Medicine, Osaka City University, Osaka 558-8585, Japan; dtsuruta@med.osaka-cu.ac.jp

**Keywords:** reactive oxygen species, free radicals, oxidative stress, skin

## Abstract

Oxygen in the atmosphere is a crucial component for life-sustaining aerobic respiration in humans. Approximately 95% of oxygen is consumed as energy and ultimately becomes water; however, the remaining 5% produces metabolites called activated oxygen or reactive oxygen species (ROS), which are extremely reactive. Skin, the largest organ in the human body, is exposed to air pollutants, including diesel exhaust fumes, ultraviolet rays, food, xenobiotics, drugs, and cosmetics, which promote the production of ROS. ROS exacerbate skin aging and inflammation, but also function as regulators of homeostasis in the human body, including epidermal keratinocyte proliferation. Although ROS have been implicated in various skin diseases, the underlying mechanisms have not yet been elucidated. Current knowledge on ROS-related and oxidative stress-related skin diseases from basic research to clinical treatment strategies are discussed herein. This information may be applied to the future treatment of skin diseases through the individual targeting of the ROS generated in each case via their inhibition, capture, or regulation.

## 1. Introduction

**Figure 1:**
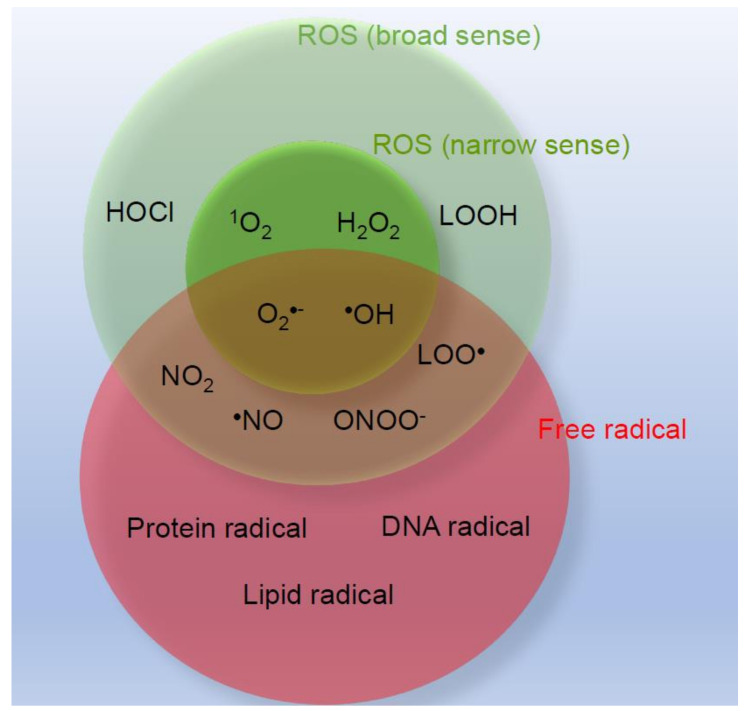
Reactive oxygen species and free radicals.

**Figure 2:**
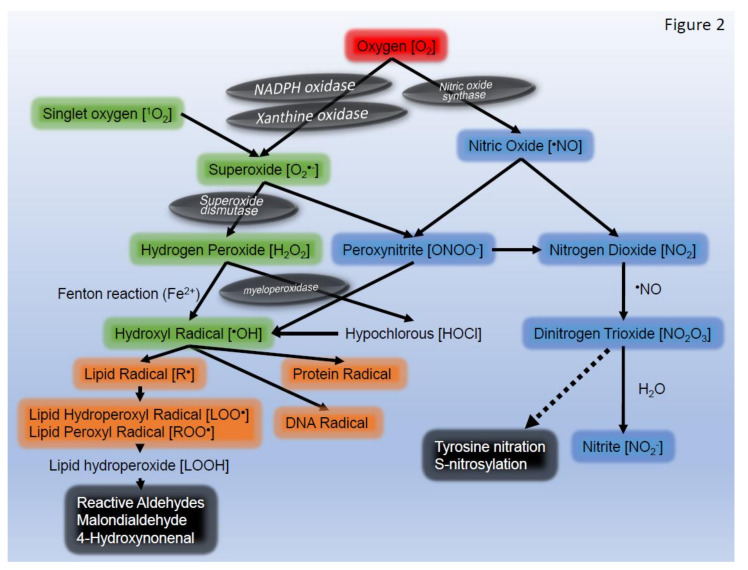
Reaction pathway of reactive oxygen species and free radicals.

Oxygen in the atmosphere is a crucial component for life-sustaining aerobic respiration in humans. Approximately 95% of oxygen is consumed as energy and ultimately becomes water; however, the remaining 5% produces metabolites called activated oxygen or reactive oxygen species (ROS), which are extremely reactive. Skin, the largest organ in the human body, is exposed to air pollutants, including diesel exhaust fumes, ultraviolet rays, food, xenobiotics, drugs, and cosmetics, which promote the production of ROS. ROS exacerbate skin aging and inflammation, but also function as regulators of homeostasis in the human body, including epidermal keratinocyte proliferation. There are four ROS: superoxide (O_2_^•−^), hydrogen peroxide (H_2_O_2_), the hydroxyl radical (^•^OH), and singlet oxygen (^1^O_2_). Nitric oxide (^•^NO) and peroxynitrite (ONOO^−^) are also included as ROS (Figure 1). These ROS are involved in complex and diverse reaction pathways (Figure 2), and sometimes form molecules and atoms with unpaired electrons called free radicals (Figure 1 and Figure 2). In the living body, free radicals exist as lipid, protein, and DNA radicals, and some ROS (O_2_^•−^, ^•^OH, ^•^NO, and ONOO^−^) are also free radicals. Oxidative stress is defined as an increase in the production of ROS and other oxidants that exceeds the antioxidant capacity. Current knowledge on ROS-related and oxidative stress-related skin diseases from basic research to clinical treatment strategies are discussed herein.

## 2. ROS Generation in Skin

ROS is generated when skin is exposed to ultraviolet rays and visible light. In the presence of photosensitizers, such as psoralen, porphyrin, and tetracycline, oxygen reaches its highest excited state via photoreaction pathways, resulting in the formation of ^1^O_2_, a strong oxidizer [1,2,3,4]. Since the lifespan of ^1^O_2_ is very short (10^−6^ to 10^−5^ s), it is difficult to detect in the living body [5]; therefore, whether ^1^O_2_ is universally produced in the living body remains unknown and its direct effects have not yet been clarified. Although ^1^O_2_ is the first ROS generated in skin exposed to visible light or ultraviolet rays, it is rapidly metabolized to O_2_^•−^, H_2_O_2_, and ^•^OH, which are considered to have various biological roles in skin. ^1^O_2_ exhibits strong oxidizing activity; however, in contrast to other ROS, its oxidation is achieved via a non-radical reaction, not a chain reaction. In other words, the reaction ends following the oxidization of the target molecule. Although H_2_O_2_ is utilized as ROS in various in vitro experiments, it is unclear whether H_2_O_2_ really has direct roles in the experiments. Other ROS, such as O_2_^•−^ and ^•^OH, cause a radical reaction, and free radicals, including lipid, protein, and DNA radicals, are generated via a chain reaction in skin. However, limited information is currently available on the free radicals generated in skin.

**Figure 3:**
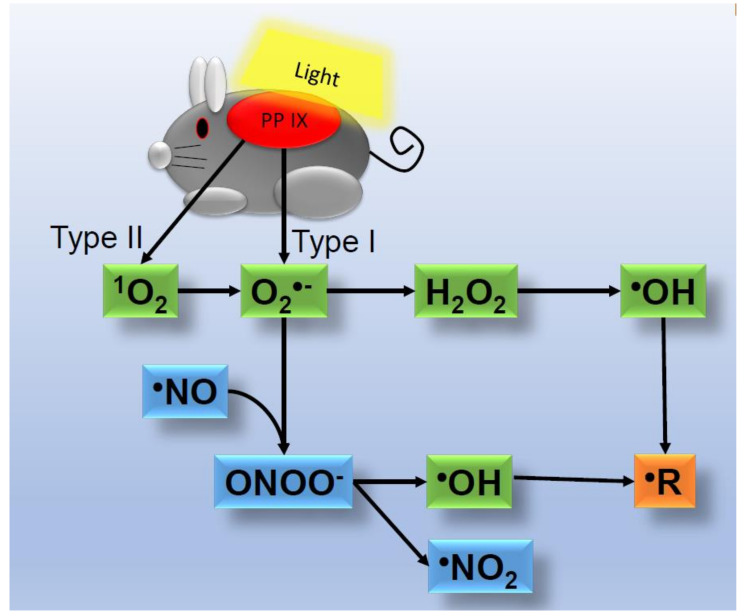
Detection of photo-induced free radicals in the skin of mice using an electron spin resonance method. Lipid radicals and ^•^OH are generated in skin treated with protoporphyrin IX (PP IX) plus natural light.

Photodynamic therapy is used to treat skin tumors and acne vulgaris [6,7,8]. Externally applied methyl aminolevulinic acid is metabolized to protoporphyrin IX (PP IX), which is a photosensitizer in highly proliferative tumor cells and *Propionibacterium acnes*. Photo-induced ROS and free radicals damage tumor cells and *P. acnes*, ultimately resulting in cell death. A previous study successfully detected photo-induced free radicals in the skin of mice using an electron spin resonance method. Furthermore, the generation of lipid radicals and oxygen-centered radicals was observed in the skin of mice treated with PP IX plus natural light (Figure 3) [3]. The findings obtained indicated that the oxygen-centered radical was ^•^OH, a metabolite of ^1^O_2_. Lipid radicals were considered to be generated by ^•^OH.

O_2_^•−^ is mainly synthesized by two enzymes, NADPH oxidase and xanthine oxidase (XO), with the exception of when ^1^O_2_ is metabolized in the body. Neutrophils and macrophages exhibit strong NADPH oxidase activity during inflammation under cutaneous infection, and a large amount of O_2_^•−^ is generated in skin [9,10]. O_2_^•−^ produced by neutrophils and macrophages is detrimental to bacteria, fungi, and viruses; however, in the case of vasculitis, it damages vascular endothelial cells [11]. Therefore, abundant amounts of NADPH oxidase-derived O_2_^•−^ are highly cytotoxic. The expression of NADPH oxidase has been detected in epidermal keratinocytes [12] and fibroblasts [13] in skin. The small amount of O_2_^•−^ produced by these cells regulates the differentiation and/or proliferation of epidermal keratinocytes and fibroblasts. Therefore, abnormal NADPH oxidase activity has been associated with a wide spectrum of skin diseases as well as aging and carcinogenesis [14].

**Figure 4:**
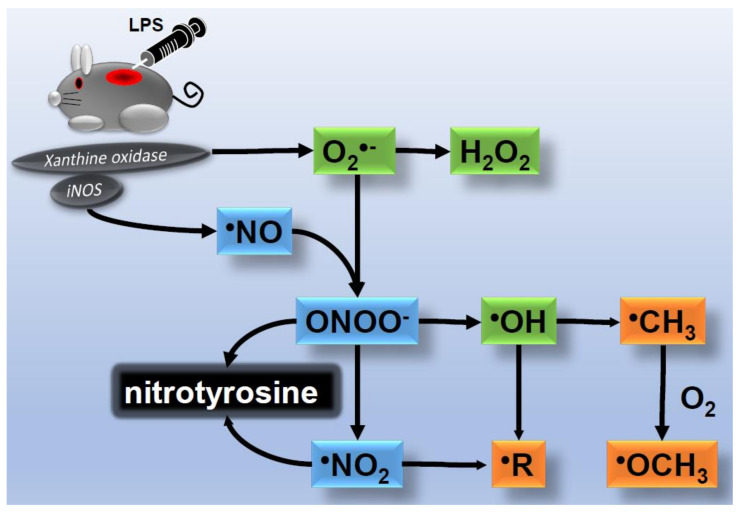
Detection of lipopolysaccharide (LPS)-induced free radicals in the skin of mice using an electron spin resonance method. The activities of XO and iNOS were increased, and ^•^NO and O_2_^•−^ were produced in the early stage of skin inflammation. Carbon-centered radicals (^•^CH_3_ and ^•^OCH_3_) and lipid radicals were generated.

XO is another enzyme that produces O_2_^•−^. XO is expressed in epidermal keratinocytes and endothelial cells [15,16]. Previous studies reported that various stimuli induced increases in XO activity, including ischemia-reperfusion after cutaneous flap surgery [17] and light irradiation [18]. Moreover, XO is required for skin inflammation induced by lipopolysaccharide (LPS), a strong stimulator of toll-like receptors, which suggests that XO and XO-induced ROS/free radicals participate in the innate immunity of skin. The free radicals generated in an LPS-induced skin inflammation mouse model were examined using electron spin resonance, and the findings obtained revealed the generation of lipid radicals 6 h after the administration of LPS (Figure 4) [19]. Since the generation of lipid radicals was suppressed by the administration of allopurinol, a specific inhibitor of XO, XO activity was considered to be elevated and O_2_^•−^ was produced in the early stage of skin inflammation. However, another study detected NADPH oxidase activity 24 h after the administration of LPS. Therefore, the source of ROS appears to depends on the inflammatory status of skin lesions.

Superoxide dismutase (SOD) is the only enzyme that eliminates O_2_^•−^ in the living body [20]. It catalyzes the reduction of O_2_^•−^ to H_2_O_2_. Three types of SOD exist in humans: manganese SOD (MnSOD) in mitochondria, copper and zinc SOD (Cu/Zn SOD) in the cytoplasm, and extracellular SOD. SOD is also present in epidermal keratinocytes and fibroblasts in skin. H_2_O_2_ is a relatively stable ROS, and since it is not a free radical, it is not as reactive as other ROS. However, in the presence of transition metals, such as iron or copper, ^•^OH, a highly reactive free radical, is generated from H_2_O_2_ via the Fenton reaction. Since transition metals are commonly present in most cells and tissues, H_2_O_2_ generates ^•^OH in vivo [21]. Therefore, H_2_O_2_ needs to be is decomposed into water by catalase, glutathione (GSH), and GSH peroxidase (GPx) as quickly as possible before it produces ^•^OH.

^•^NO is mainly synthesized by three types of NO synthase (NOS): the neural type (nNOS), inducible type (iNOS), and endothelial type (eNOS). All three NOS types are expressed in epidermal keratinocytes in skin, iNOS is mainly present in fibroblasts, and eNOS is the predominant type in vascular endothelial cells. Although the roles of nNOS and eNOS have not yet been elucidated, they have been proposed to function in skin homeostasis, such as epidermal proliferation, keratinization, and the regulation of dermal blood flow. Previous studies demonstrated that iNOS synthesized a large amount of ^•^NO during skin inflammation caused by ultraviolet exposure, psoriasis vulgaris, systemic lupus erythematosus, and wound healing [22,23,24,25]. iNOS activity was also shown to be significantly elevated in an LPS-induced inflammatory skin disease mouse model (Figure 4) [19]. On the other hand, a long-term treatment with a high concentration of glucose reduced the expression and activity of iNOS, whereas it increased the activities of nNOS and eNOS in epidermal keratinocytes [26,27], suggesting that iNOS activity is decreased in the skin of diabetic patients. Since iNOS is required for skin wound healing and inflammation, diabetic patients are susceptible to skin complications, including bacterial and fungal infections and intractable ulcers.

In the presence of a large amount of ^•^NO, O_2_^•−^ may form ONOO^−^ which is further decomposed into nitrogen dioxide radicals (^•^NO_2_) and ^•^OH. ^•^NO, ONOO^−^ and ^•^NO_2_ collectively nitrate tyrosine residues of proteins to form nitrotyrosine [28]. Tyrosine residues in proteins are the targets of phosphorylation/dephosphorylation by tyrosine kinase/phosphatase and are closely involved in intracellular signal transduction. Phosphorylation does not occur when tyrosine residues are nitrated. Therefore, the formation of nitrotyrosine has been suggested to suppress phosphorylation signals [29]. XO and iNOS were both required for the formation of lipid radicals and nitrotyrosine in an LPS-treated inflammatory skin mouse model [19]. Since the administration of a metal adsorbent (desferal) did not reduce the formation of lipid radicals, ^•^OH was not a metabolite of H_2_O_2_, but was generated via ONOO^−^ with the simultaneous formation of nitrotyrosine.

Difficulties are associated with measuring the production of ROS/RNS or free radicals in the skin or blood of patients with skin diseases. As an alternative, methods to detect ROS-related metabolites, ROS-generating enzymes, and antioxidants has been reported. The oxidation products of lipids, proteins, and DNA in blood, plasma, tissue, and urine have been measured collectively as ROS/RNS or free radicals. The concentrations of malondialdehyde (MDA), lipid hydroperoxides, thiobarbituric acid reactive substances (TBARS), isoprostanes, protein carbonyls, tyrosine products, methionine sulfoxidation, and 8-hydroxy-2′-deoxyguanosine (8-OHdG) have been investigated to establish whether ROS promote the generation of these oxidation products. MDA may be one of major products of lipid oxidation [30], and 8-OHdG has been utilized as a marker of DNA oxidation [31]. Furthermore, nitrite and nitrate have been measured as direct metabolites of ^•^NO [32].

## 3. Roles of ROS Generated in Skin

Since ROS and free radicals are highly reactive and unstable, their direct roles in skin remain unclear. However, ROS produce relatively stable oxidants in vivo, including 4-hydroxy-2-nonenal and MDA. These oxidants change the structures of proteins, induce cell apoptosis, and regulate the release of inflammatory cytokines. ROS also trigger various biological responses through the activation of transcription factors, such as activator protein 1 (AP-1), mitogen-activated protein kinase (MAPK), and nuclear factor kappa B (NF-κB). HB-EGF was previously shown to activate eNOS in order to produce vascular endothelial growth factor via PI3 kinase and MAPK in human keratinocytes [33]. Lipid peroxidation also induced the expression of vascular endothelial growth factor in human keratinocytes [34]. Angiotensin II increased EGF receptor expression levels via the formation of ROS in human keratinocytes [35]. Furthermore, ROS activated Akt in human dermal fibroblasts [36]. Ferroptosis is a newly recognized form of programmed cell death that differs from the other forms of cell death, including apoptosis, necroptosis, and pyroptosis [37]. It is characterized by ROS-induced lipid oxidation and iron overload. Recent findings have suggested that ferroptosis is involved in the pathogenesis of psoriasis, skin cancers, and collagen diseases [38,39,40].

## 4. Antioxidants in Skin Tissue

**Figure 5:**
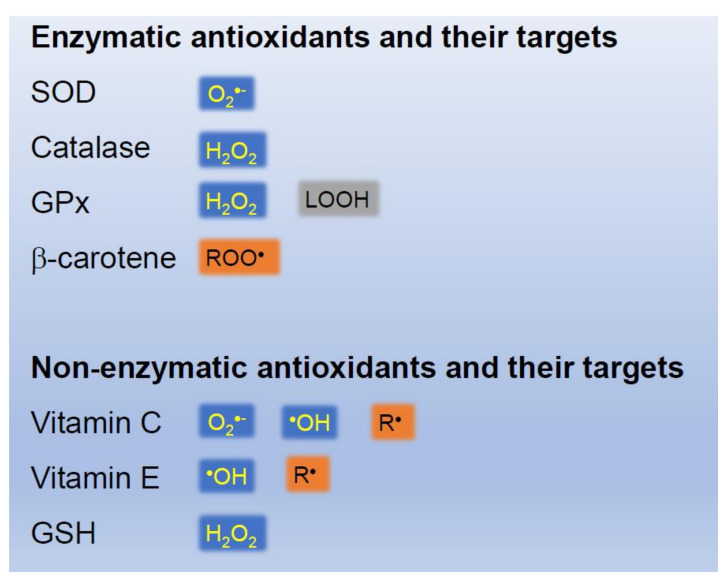
Examples of enzymatic and non-enzymatic antioxidants.

Many antioxidants eliminate ROS from the living body. There are essentially two types of antioxidants, enzymatic and non-enzymatic (Figure 5). SOD is a typical enzymatic eliminator of O_2_^•−^. Catalase and GPx decompose and eliminate H_2_O_2_. Catalase is one of the crucial antioxidant enzymes that strongly mitigates oxidative stress by decomposing cellular H_2_O_2_ into water. A deficiency in or the malfunction of catalase has been implicated in the pathogenesis of many age-associated degenerative diseases, such as diabetes mellitus, hypertension, vitiligo, Alzheimer’s disease, and cancer [41]. GPx uses GSH to reduce H_2_O_2_ [42]. Eight GPx have been identified to date in mammals (GPx1–GPx8) [43], some of which react with lipid hydroperoxides.

**Figure 6:**
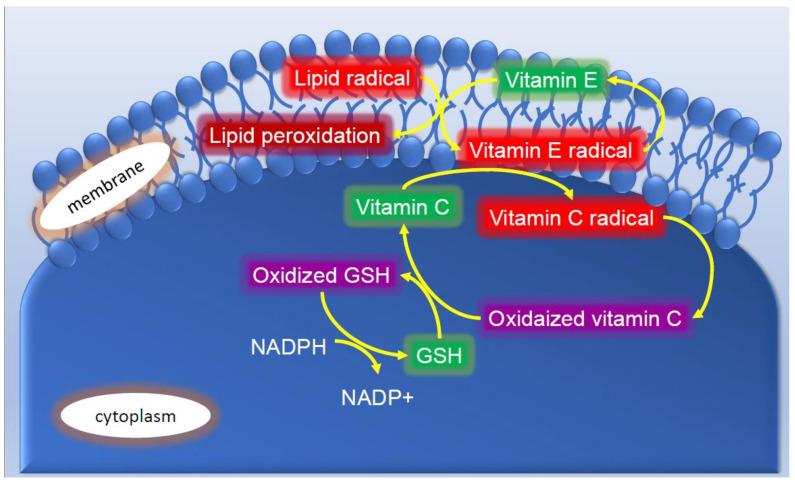
Reaction mechanisms of non-enzymatic antioxidants.

Well-known non-enzymatic antioxidants include vitamin C, vitamin E, GSH, and β-carotene [44]. However, these antioxidants may only react with H_2_O_2_. Vitamin E suppresses lipid radicals in cell membranes. Vitamin C reacts with O_2_^•−^ and ^•^OH in the cytoplasm to become a vitamin C radical, which is then oxidized. Vitamin E radicals are restored to vitamin E. GSH prevents the oxidation of vitamin C. Therefore, a single antioxidant may not be sufficient to eliminate ROS and free radicals (Figure 5 and Figure 6). Vitamin C has been shown to enhance iNOS activity under specific conditions [45]. N-acetylcysteine (NAC) is a precursor of GSH. Polyphenols in green tea and wine may capture ROS and free radicals generated in skin [46].

## 5. ROS and Skin Diseases

**Figure 7:**
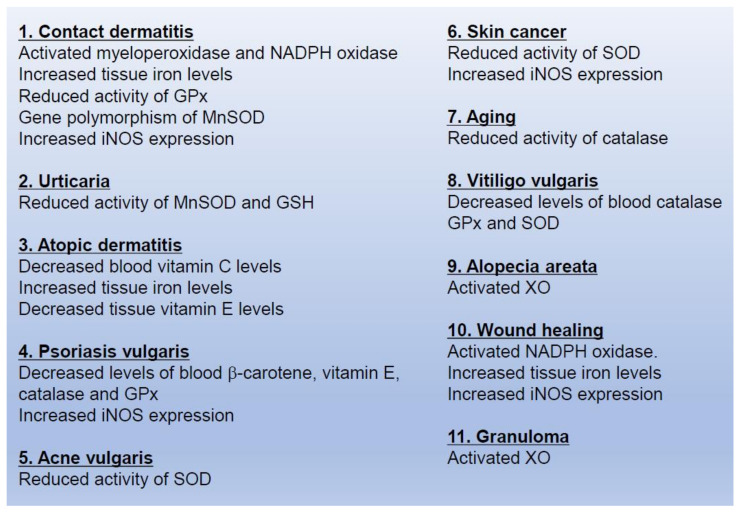
Representative reported abnormalities in the production of reactive oxygen species in skin diseases.

Difficulties are associated with the direct measurement of ROS generated in the skin and blood of patients with skin diseases. Previous studies reported both increases or decreases in the metabolites of ROS, ROS generators, and antioxidants in skin diseases, with abnormal ROS generation being suggested to play a role in their pathogenesis (Figure 7).

### 5.1. Contact Dermatitis

The abnormal metabolism of ROS has been detected in the skin of patients with contact dermatitis. The dominant sources of ROS appear to be myeloperoxidase and NADPH oxidase in the skin lesions of contact dermatitis [47]. Since iron concentrations are elevated and/or GPx activity is reduced, ^•^OH is easily generated in the skin tissue of individuals with a positive nickel patch test [48]. The incidence of contact dermatitis due to p-phenylenediamine, a component of hair dye, has been associated with gene polymorphisms in MnSOD in middle-aged women [49]. NAC, a precursor of GSH, reportedly suppresses contact dermatitis by inhibiting the phosphorylation of tyrosine residues in CD14-positive antigen-presenting cells when it is sensitized with p-phenylenediamine [50]. A significant increase in iNOS expression levels was immunohistochemically detected in the skin lesions of patients with contact dermatitis [51]. 2.4-Dinitrofluorobenzene has been shown to induce the expression of iNOS in keratinocytes and Langerhans cells [52]. Furthermore, it irreversibly inhibited mammalian thioredoxin reductase with the formation of ROS [53].

### 5.2. Urticaria

The etiology of urticaria has not yet been clarified. Although recent findings implicate ROS [54,55], the role of oxidative stress in the pathogenesis of urticaria is controversial. Mast cells and basophils are capable of generating O_2_^•−^ to regulate their activation, including the release of their characteristic chemical mediators [56]. The activities of MnSOD and GSH and the level of MDA were previously shown to be markedly elevated in the skin lesions of a patient with urticaria, whereas CuZnSOD activity was unchanged [55]. Moreover, no significant differences were detected between unaffected skin from urticaria patients and that from healthy controls. A previous study demonstrated that the production of ROS and plasma SOD activity were significantly elevated in the peripheral blood of a patient with urticaria and treatment with desloratadine caused a reduction in these parameters [57]. Furthermore, no significant differences were observed in antioxidant enzyme activities in plasma or erythrocytes between urticaria patients and healthy controls [58].

### 5.3. Atopic Dermatitis

The dense infiltration of lymphocytes, monocytes, and eosinophils has been histologically demonstrated in the skin lesions of patients with atopic dermatitis. These inflammatory cells may produce a large amount of ROS, including O_2_^•−^ and ^•^NO, as well as cytokines [59]. Therefore, ROS-related markers are elevated in patients with atopic dermatitis [60]. Patients with atopic dermatitis also have reduced vitamin C blood levels and elevated iron tissue levels in skin lesions. Therefore, ^•^OH and lipid radicals appear to be easily generated in these patients and remain in tissues [61]. The concentration of vitamin E was previously shown to be lower in skin lesions than in healthy skin, whereas that of lipid peroxide was higher. Since NAC, an antioxidant, strongly suppresses the Th2-mediated IL-4 response and mildly suppresses the secretion of IL-5 and INF-γ, it is useful for the treatment of Th2-related diseases, such as atopic dermatitis [62]. Previous studies demonstrated that NAC improved the epidermal barrier function of patients with atopic dermatitis [63,64].

### 5.4. Psoriasis Vulgaris

The excessive production of cytokines by epidermal keratinocytes and activation of neutrophils and lymphocytes in the skin lesions of psoriasis results in the generation of abundant amounts of ROS and free radicals. ROS-related markers were previously found to be elevated in patients with psoriasis [60]. Furthermore, marked increases were observed in the blood MDA levels of patients with psoriasis, whereas blood antioxidant levels, including β-carotene, vitamin E, catalase, and GPx, were reduced [65]. Although iNOS was overexpressed in the skin lesions of patients with psoriasis [23], disease symptoms were not attenuated by the topical application of L-NAME, a pan-NOS inhibitor [66]. L-NAME has been shown to inhibit eNOS and nNOS, activities which suppresses the production of O_2_^•−^. L-NAME may not have been effective in the treatment of psoriasis because O_2_^•−^ concentrations are elevated in the skin lesions of these patients due to the inhibition of eNOS and nNOS activities. Therefore, the combination of SOD and/or catalase with L-NAME may represent a novel treatment strategy for psoriasis. Moreover, although macrophages are regarded as the main producers of ROS, they also express iNOS, the mannose receptor, and klotho in the skin lesions of patients with psoriasis [67,68], indicating that macrophages do not simply produce ^•^NO by activating iNOS. Therefore, further studies are needed to clarify the roles of ROS and/or macrophages in the pathogenesis of psoriasis.

### 5.5. Acne Vulgaris

In the lesions of inflammatory acne vulgaris, a large amount of O_2_^•−^ was shown to be generated by neutrophils infiltrating hair follicles [69] and SOD activity was reduced [70]. Furthermore, the ratio of linoleic acid was low, whereas that of palmitic acid was high. O_2_^•−^ levels were reduced by linoleic acid, but not by palmitic acid. Therefore, tissue damage was promoted in the skin lesions of acne vulgaris [71]. *P. acnes* produces porphyrin in the sebaceous glands, indicating the generation of ^1^O_2_ by visible light. Squalene, a lipid localized in the sebaceous glands, captures ^1^O_2_ and suppresses the peroxidation of other proteins and lipids in skin; therefore, the oxidation of squalene exacerbates acne vulgaris [72]. Vitamin E and squalene concentrations in the sebaceous glands of the face are sufficient to protect important skin lipids, proteins, and DNA against the effects of light exposure. Minocycline and doxycycline have been shown to suppress the production of ROS derived from polymorphonuclear leukocytes, and, thus, are considered to be effective in the treatment of acne vulgaris [73]. Benzoyl peroxide and photodynamic therapy are also used to treat acne vulgaris because they damage *P. acnes* and sebaceous cells through the production of ROS [74,75].

### 5.6. Skin Cancer

The mechanisms underlying the development of malignant skin tumors (i.e., squamous cell carcinoma, basal cell carcinoma, and malignant melanoma) include ultraviolet-related DNA damage, tumor-associated gene expression, and dysfunctional intracellular signal transduction. ROS and free radicals are considered to play a role in these mechanisms. They have been implicated in all three stages of carcinogenesis, namely, initiation, promotion, and progression. Antioxidant levels, such as SOD, were previously found to be reduced in the blood of patients with actinic keratosis and basal cell carcinoma [76]. The abnormal activation of intracellular signal transduction by ROS was detected in malignant melanoma. The prognosis of patients with malignant melanoma expressing iNOS is known to be poor [77]. Polyphenols down-regulate the cell cycle of tumor cells by capturing ROS and free radicals, suppressing cell proliferation, and inducing apoptosis. Klotho is an anti-aging factor that may restore antioxidant molecules, including SOD [78], and its expression was found to be down-regulated in the epidermal keratinocytes of non-skin cancers [79,80].

### 5.7. Aging

Skin aging is divided into natural aging and photoaging. The catalase activity of skin fibroblasts decreases with natural aging, while H_2_O_2_ concentrations increase. Matrix metalloproteinase-1 is subsequently activated, which ruptures dermal collagen fibrils [81]. In photoaging, ^1^O_2_ generated by photo exposure and other ROS metabolites of ^1^O_2_ and free radicals have been shown to directly damage the DNA and lipids of epidermal keratinocytes and skin fibroblasts. ROS and free radicals activate transcriptional factors, including NF-κB and AP-1. As a result, collagen fibers are degraded by inflammatory cytokines and enzymes. Protease-activated receptor 2 has been reported to induce ROS-related inflammation via the Akt-NF-κB pathway with the suppression of FoxO6 during skin photoaging [82].

In addition, pigmentation (stains) caused by UV irradiation may be attributed to the promotion of melanin synthesis by O_2_^•−^ and H_2_O_2_ [83]. UV exposure also produces ^•^NO and promotes melanin synthesis.

### 5.8. Vitiligo Vulgaris

Catalase, GPx, and SOD activities were previously reported to be decreased in the blood of patients with vitiligo vulgaris, while MDA levels were elevated in the advanced stage [84]. However, the expression levels of SOD and GPx have been shown to increase regardless of the accumulation of MDA in skin lesions [85]. An abnormal immune response in addition to the excessive generation of ROS in the body have been implicated in the development of vitiligo vulgaris.

### 5.9. Alopecia Areata

Although the activities of SOD and GPx were increased in the skin lesions of alopecia areata, TBARS levels remained unchanged [86]. In contrast, MDA and nitrite levels and XO activity were elevated in the serum of these patients [87,88], whereas the activity of SOD was low [87,89].

### 5.10. Wound Healing

Wound healing consists of multiple processes, and ROS are generated in each process. During the inflammatory phase, neutrophils infiltrate the wound site and generate a large amount of O_2_^•−^ derived from NADPH oxidase. Bleeding increases iron concentrations and induces the generation of ^•^OH. ONOO^−^ is also produced because iNOS in various cells at the wound site is activated. Although the role of these ROS currently remains unclear, iNOS activity is low in diabetic ulcers [90], and iron is deposited in the tissue of skin ulcers due to stasis dermatitis [91]. Therefore, the abnormal generation of ROS is considered to cause intractable skin ulcers. Hydrogels containing antioxidants have been shown to improve the activities of SOD and GPx and promote wound healing [92]. Carboxymethyl chitosan hydrogel, a ROS eliminator, is useful for healing burn wounds [93].

### 5.11. Granuloma

Epithelioid cells and giant cells have been histologically detected in the lesions of sarcoidosis and foreign body granuloma. These cells are considered to produce XO-derived O_2_^•−^. Allopurinol is a popular therapeutic agent for gout, but is also a specific inhibitor of XO. Previous studies reported that the administration of allopurinol attenuated sarcoidosis and foreign body granuloma [94,95].

### 5.12. Other Skin Diseases

ROS have been implicated in the development of various diseases, such as collagen diseases, in addition to the diseases described above. Raynaud’s phenomenon has been proposed in systemic scleroderma because excess ROS produced by fibroblasts around blood vessels induce abnormalities in blood vessel functions. Furthermore, blood SOD activity was found to be elevated in individuals with Raynaud’s phenomenon for a long period of time, which functioned as a defense reaction [96]. Numerous studies have suggested the involvement of ROS in lichen planus based on ROS markers [97]; however, the origin and involvement of ROS in its pathogenesis remain unclear. Similarly, the accumulation of acrolein, a lipid peroxidation product, has been detected in the skin of patients with small-vessel vasculitis [98], and a relationship between ROS and the severity of vessel damage has been implied [99]. A similar relationship has been suggested for seborrheic dermatitis [100]. The potential roles of increased lipid peroxidation and peroxidation, and decreased antioxidant levels in pemphigus vulgaris have been reported [101]. Since ROS levels are increased and antioxidant potential is decreased in rosacea [102], some therapeutic agents are utilized as antioxidant drugs. Allopurinol is useful for the treatment of perforating skin diseases, such as granulomatous skin diseases, and the involvement of XO-derived O_2_^•−^ in these diseases has been indicated [103].

## 6. Concludings

In this review, some examples of free radical generation in skin were introduced: photo-induced and LPS-induced free radical production. Based on these observations together with other reports concerning ROS-related skin diseases, the dynamics of free radical and ROS in skin pathophysiology might be clarified a little. Although antioxidant drugs are now utilized for the treatment of various skin diseases, the effects of these drugs are limited. The regulation of free radical and ROS has been implicated in the development of various skin diseases; however, the underlying mechanisms have not yet been elucidated in detail, and proper usage of antioxidant drugs is still unknown now. In addition, there lacks studies concerning physiological roles of free radicals and ROS in skin homeostasis, and the information about duration of the generation, action and ratio of free radicals and ROS is still unclear in skin diseases. Most in vitro studies have demonstrated the acute transient effects of free radicals and ROS, and only a few studies have shown the chronic effects of them that appear to be different from the acute effects. We could not find enough references clarifying these issues in in vivo experiments and human skin diseases, and almost all studies have demonstrated acute effects of free radicals and ROS/RNS in in vivo experiments and human skin diseases. We speculate that it is because the amount of physiologically or constitutively (chronically) produced free radicals and ROS/RNS is too small to detect in in vivo experiments and human skin diseases. The information obtained in future studies may be applied to the treatment of skin diseases through the individual targeting of the ROS generated in each case via their inhibition, capture, or regulation.
